# A meta-analysis of genome-wide gene expression differences identifies promising targets for type 2 diabetes mellitus

**DOI:** 10.3389/fendo.2022.985857

**Published:** 2022-08-16

**Authors:** Tao Huang, Bisma Nazir, Reem Altaf, Bolun Zang, Hajra Zafar, Ana Cláudia Paiva-Santos, Nabeela Niaz, Muhammad Imran, Yongtao Duan, Muhammad Abbas, Umair Ilyas

**Affiliations:** ^1^ Henan Provincial Key Laboratory of Pediatric Hematology, Children’s Hospital Affiliated to Zhengzhou University, Zhengzhou University, Zhengzhou, China; ^2^ Medical School, Huanghe Science and Technology University, Zhengzhou, China; ^3^ Riphah Institute of Pharmaceutical Sciences, Riphah International University, Islamabad, Pakistan; ^4^ Department of Pharmacy, Islamabad, Pakistan; ^5^ School of Pharmacy, Shanghai Jiao Tong University, Shanghai, China; ^6^ Department of Pharmaceutical Technology, Faculty of Pharmacy, University of Coimbra, Coimbra, Portugal; ^7^ REQUIMTE/LAQV, Group of Pharmaceutical Technology, Faculty of Pharmacy, University of Coimbra, Coimbra, Portugal; ^8^ Department of Pharmacy, Sarhad University of Science and Technology, Peshawar, Pakistan

**Keywords:** type 2 diabetes mellitus, gene ontology, differential expression analysis, R programming, system biology

## Abstract

**Aims/introduction:**

Due to the heterogeneous nature of type 2 diabetes mellitus and its complex effects on hemodynamics, there is a need to identify new candidate markers which are involved in the development of type 2 diabetes mellitus (DM) and can serve as potential targets. As the global diabetes prevalence in 2019 was estimated as 9.3% (463 million people), rising to 10.2% (578 million) by 2030 and 10.9% (700 million) by 2045, the need to limit this rapid prevalence is of concern. The study aims to identify the possible biomarkers of type 2 diabetes mellitus with the help of the system biology approach using R programming.

**Materials and methods:**

Several target proteins that were found to be associated with the source genes were further curated for their role in type 2 diabetes mellitus. The differential expression analysis provided 50 differentially expressed genes by pairwise comparison between the biologically comparable groups out of which eight differentially expressed genes were short-listed. These DEGs were as follows: *MCL1*, *PTX3*, *CYP3A4*, *PTGS1*, *SSTR2*, *SERPINA3*, *TDO2*, and *GALNT7*.

**Results:**

The cluster analysis showed clear differences between the control and treated groups. The functional relationship of the signature genes showed a protein–protein interaction network with the target protein. Moreover, several transcriptional factors such as DBX2, HOXB7, POU3F4, MSX2, EBF1, and E4F1 showed association with these identified differentially expressed genes.

**Conclusions:**

The study highlighted the important markers for diabetes mellitus that have shown interaction with other proteins having a role in the progression of diabetes mellitus that can serve as new targets in the management of DM.

## Background

Type 2 diabetes mellitus is known to be a life-long disease that is linked to disturbances in glucose levels in the blood. This disease acts as a silent killer that is affecting a wider population of the world. The need to develop potential target hypoglycemic agents as well as new biological candidates and possible treatment strategies has been emphasized in order to combat this disease (1). Type 2 diabetes mellitus has become prevalent day by day, and at present, doctors have an increasing number of patients who suffer from this disease (2). Both insulin resistance and defects in the secretion of insulin have shown a role in the pathogenesis of type 2 diabetes mellitus (3). Individuals suffering from type 2 diabetes mellitus encounter microvascular complications such as retinopathy, nephropathy, and neuropathy and also macrovascular complications including cardiovascular comorbidities, contributing to the development of hyperglycemia and insulin resistance (metabolic) syndrome (4).

The system biology approach aids in understanding integrative physiological responses through the incorporation of experimental and computational approaches. It provides a powerful foundation from which to address complex scientific questions (5). The R software is a recent, functional programming language that creates high-quality graphical productivity, and all the stages of a study, from analysis to publication, can be undertaken within R (6). R is a very powerful statistical tool and is especially used for data manipulation, calculations, and plots (7). *In-silico* or docking studies are those research tools that are “run on or performed on a computer or done *via* computer simulation.” Drug designing and discovery involves the development of new potential targets for drug molecules through computer simulation, and it is a rapidly emerging trend nowadays (8). Several mechanisms are under the control of selective gene expressions in molecular biology. The difference in the expression between normal tissue and diseased tissue aids in identifying the disease mechanism. The dysregulation of genes and pathways in normal tissue occasionally leads to a disease state that after recognition can guide in making therapy decisions. Functional enrichment analysis and gene ontology studies provide the affiliation data of such proteins and the collaborating proteins that help in isolating these topographies (9).

The main aim of this study is the screening of potential biomarkers for type 2 diabetes mellitus for the better management of this disease. The target-based treatment strategies have proven fruitful due to their site-specific efficacy and reduced side effects (10). For the development of target-based drugs, the system biology approach has been found useful. This approach aids in the identification of important gene signatures that are known to play an essential role in disease etiology. Targeting these genes can slow down the pace of the progression of DM. Therefore, type 2 diabetes mellitus differentially expressed genes have been identified and short-listed. The role of these genes has been curated and the gene ontology and annotation of these genes will also be studied. Moreover, a gene network analysis of signature genes with other potential interacting proteins will also be mapped to further clarify the role of these genes in the progression of type 2 diabetes mellitus. The miRNA target hits for these identified DEGs will also be predicted. This will aid particularly in targeting these specific signature genes or associated genes involved in the pathogenesis of this disease, thus opening new treatment strategies.

## Methods

### Derivation of the GEO datasets

In this study, the potential type 2 diabetes mellitus targets were identified through a differential screening method. Ten datasets of type 2 diabetes mellitus were retrieved using the NCBI database Gene Expression Omnibus. The dataset used was “organism: Homo sapiens” and “experiment type: expression profiling by array”. The Affymetrix GeneChip Human Genome U133 Plus 2.0 Array was used (Affymetrix, Inc., Santa Clara, CA, 95051, USA). All the cDNA datasets included various information including platform, GEO accession number, number of samples, sample type, and genetic expression data. The differentially expressed genes were identified using the array platform and the annotation platform hgu133plus2 probes. For the computational analysis, the R programming and the Bioconductor packages, affy QC Report, affy, annotate, AnnotationDbi, limma, Biobase, AffyRNAdegradation, hgu133plus2cdf, and hgu133a2cdf, were used (11).

### Processing of cDNA datasets

The phenodata files for each dataset having the “.cel format” were prepared for initial preprocessing. For normalization, the Bioconductor Array Quality Metrics package was employed in R programming to a median expression level for each gene (version 3.1.3). After the normalization, the second step was the background correction for perfect match (pm) and mismatch (mm) using the robust multiarray analysis (RMA). This method reduces the local noise present in the background. Afterward, the RMA algorithm summarization was performed to measure the averages among the probes in a probe set and to attain the summary of intensities.

The degradation of RNA was analyzed in order to determine the quality of RNA in these microarray datasets measured by using the AffyRNAdegradation package of Bioconductor. Lastly, the differentially expressed genes in each dataset were recognized using the pairwise comparison. The Benjamini–Hochberg approach was applied for multiple testing correction in order to shortlist the DEGs. The shortlisting of DEGs was done on the basis of the resulting scores and *p*-values that were then ranked. The cutoff values for shortlisting the genes were *p*-value ≤0.05, false discovery rate (FDR)  <0.05, and absolute log fold change logFC  >1 (11).

### Data mapping and screening

The differentially expressed genes obtained after shortlisting were further mapped to confirm their role in type 2 diabetes mellitus. This was done with the help of varied data sources such as PubMed (http://www.ncbi.nlm.nih.gov/pubmed), Medical Subject Headings (MeSH) (http://www.ncbi.nlm.nih.gov/mesh), Online Mendelian Inheritance in Man (OMIM) (http://www.ncbi.nlm.nih.gov/omim), and PubMed Central (PMC) databases (http://www.ncbi.nlm.nih.gov/pmc). Biomedical text mining was done to filter the potential genes specific to the disease.

### Cluster analysis

The expression values present in each dataset were evaluated for cluster analysis using the CIMminer tool applying the absolute Pearson correlation analysis. A significant variation in the gene expression levels among the control and treated samples was observed in the cluster analysis (12).

### Protein–protein analysis and network construction

A network analysis was performed to study the interaction pattern of each protein with other genes having variable physiological functions in the pathological conditions when compared to normal signifying a protein–protein interaction of the signature genes. The Search Tool for the Retrieval of Interacting Genes/Proteins (STRING) and the Human Annotated and Predicted Protein Interaction (HAPPI) databases helped in evaluating the interaction of proteins with each other having a confidence score of 0.999. The Cytoscape software (version 3.2.1, Temple Place, Suite 330, Boston, MA 02111-1307, USA) was utilized to visualize and analyze the molecular interactions of signature genes with the target genes. Various databases including OMIM, MeSH, and PMC were used to recognize the role of target genes in type 2 diabetes mellitus, and the associated gene signatures cause a pathological phenotype by dysregulation. The online tool Database for Annotation Visualization and Integrated Discovery (DAVID) and the functional enrichment tool FunRich were used to study the physiological functions of signature genes including functional annotation and gene ontology (11, 12).

### miRNA target prediction

miRNAs have shown a potential influence on gene targets, and hence, the prediction of miRNAs has proven beneficial in better understanding disease etiology. Several diseases are linked to the dysregulation of miRNAs causing disruption of signaling pathways. Therefore, RNA prediction was done using the miRDB target predictor (www.mirdb.org). It is an online database for the target prediction and functional annotation of miRNAs. The target score (≤99) was used to select miRNAs.

## Results

### Identification of differentially expressed genes

**Figure 1:**
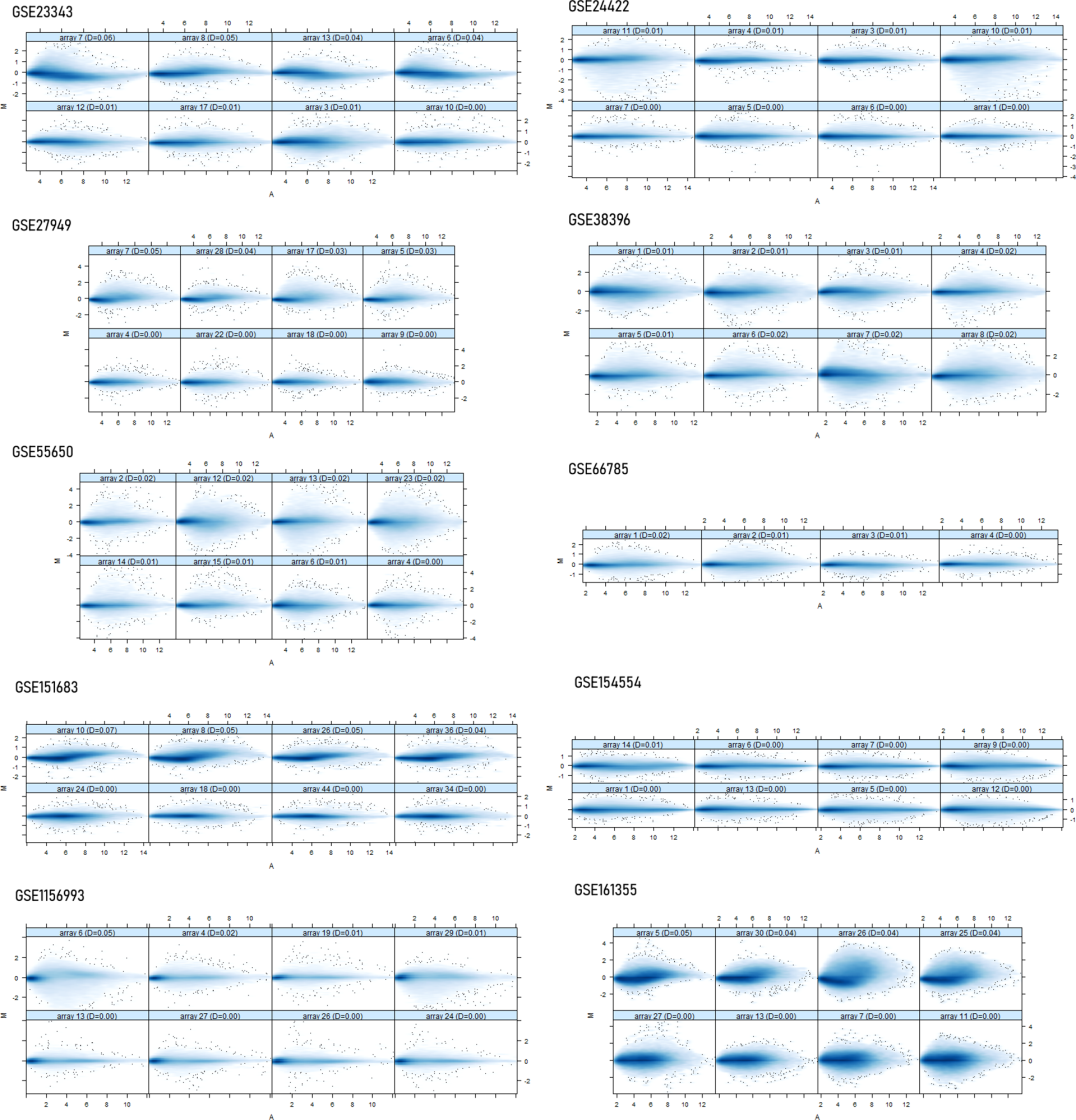
The normalization and the quality array metrics are represented by the MA plots showing the log intensity ratio (M) vs. log intensity averages (A). Usually, the mass of distribution in the MA plot is likely to be intense along the M = 0 axis.

**Figure 2:**
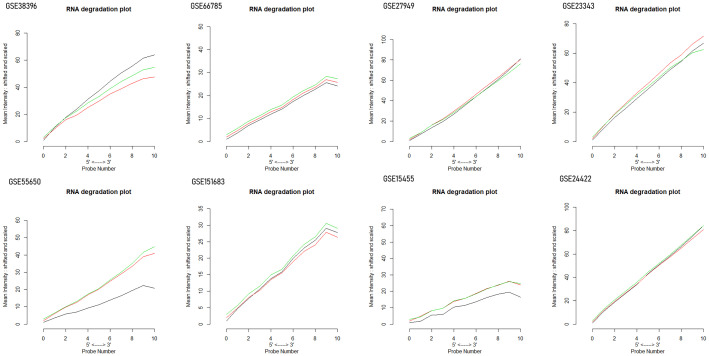
RNA degradation plots obtained by the plot AffyRNAdeg package of R demonstrating RNA quality and severity of degradation.

Ten datasets of type 2 diabetes mellitus having the “.cel format” were retrieved from the NCBI GEO database. Each dataset had a size of ArrayBatch object 1,164 × 1,164 features with associated affyIDs (**Table 1**). The normalization and background correction was done using quantile normalization in order to avoid systematic variation. The MA plot was obtained after normalization showing the quality of arrays in each dataset defining the probe level data (**Figure 1**). The degradation of RNA and the severity were represented by the AffyRNAdeg plot of the affy package of R (**Figure 2**). The AffyRNAdeg package provided the single summary statistic for each array in the batch (**Additional File 1**; **Table S1**). **Additional File 2**; **Table S2** enlists the databases, tools, and software used in this study.

**Table 1:** cDNA datasets obtained from the GEO databases.

Dataset accession no.	Total samples	Tissues	Species	Condition/type	Platform	Size of arrays	affyIDs	References
**GSE23343**	17	Muscle	*Homo sapiens*	Control vs. treated	GPL570 [HGU133_Plus_2] Affymetrix Human Genome U133 Plus 2.0 Array	1,164 × 1,164	54675	(13)
**GSE24422**	24	Adipose and stromal	*Homo sapiens*	Control vs. treated	GPL570 [HGU133_Plus_2] Affymetrix Human Genome U133 Plus 2.0 Array	1,164 × 1,164	54675	(14)
**GSE27949**	33	Adipose	*Homo sapiens*	Control vs. treated	GPL570 [HGU133_Plus_2] Affymetrix Human Genome U133 Plus 2.0 Array	1,164 × 1,164	54675	(15)
**GSE38396**	8	Skin	*Homo sapiens*	Control vs. treated	GPL570 [HGU133_Plus_2] Affymetrix Human Genome U133 Plus 2.0 Array	1,164 × 1,164	54675	(16)
**GSE55650**	23	Muscle	*Homo sapiens*	Control vs. treated	GPL570 [HGU133_Plus_2] Affymetrix Human Genome U133 Plus 2.0 Array	1,164 × 1,164	54675	(17)
**GSE66785**	4	Muscle	*Homo sapiens*	Control vs. treated	GPL570 [HGU133_Plus_2] Affymetrix Human Genome U133 Plus 2.0 Array	1,164 × 1,164	54675	(18)
**GSE151683**	46	Blood	*Homo sapiens*	Control vs. treated	GPL570 [HGU133_Plus_2] Affymetrix Human Genome U133 Plus 2.0 Array	1,164 × 1,164	54675	(19)
**GSE154554**	16	Muscle	*Homo sapiens*	Control vs. treated	GPL570 [HGU133_Plus_2] Affymetrix Human Genome U133 Plus 2.0 Array	1,164 × 1,164	54675	(20)
**GSE156993**	30	Blood	*Homo sapiens*	Control vs. treated	GPL570 [HGU133_Plus_2] Affymetrix Human Genome U133 Plus 2.0 Array	1,164 × 1,164	54675	(21)
**GSE161355**	33	Brain	*Homo sapiens*	Control vs. treated	GPL570 [HGU133_Plus_2] Affymetrix Human Genome U133 Plus 2.0 Array	1,164 × 1,164	54675	(22)

### Screening of differentially expressed genes

About 50 DEGs were obtained in each dataset through differential expression analysis by pairwise comparison among biologically similar groups. From the 10 datasets, 20 DEGs were selected. This was done on the basis of *p*-value and logFC factors. These 20 DEGs were curated to eight common signature genes that were considered as candidate markers for type 2 diabetes mellitus (**Additional File 3**; **Table S3**).

### Mapping of DEGs

The curation and mapping of DEGs was done using different databases such as PubMed, OMIM, MeSH, and PMC giving the following potential type 2 diabetes mellitus-associated genes: *MCL1*, *PTX3*, *CYP3A4*, *PTGS1*, *SSTR2*, *SERPINA3*, *TDO2*, and *GALNT7*. The function of these signature genes was curated and counted. **Table 2** shows the differentially expressed type 2 diabetes mellitus-associated genes which were curated.

**Table 2:** List of differentially expressed type 2 diabetes-related signature genes after curation.

S. no.	Probe ID	Gene ID	UniProt ID	PMC count	Protein name	Reference link
**1**	200797_s_at	MCL1	MCL1_HUMAN	325	BCL2 family apoptosis regulator (MCL1)	https://www.ncbi.nlm.nih.gov/pmc/?term=MCL1+in+type+2+diabetes
**2**	205128_x_at	PTGS1	PGH1_HUMAN	220	Prostaglandin-endoperoxide synthase 1 (PTGS1)	https://www.ncbi.nlm.nih.gov/pmc/?term=PTGS1++in+type+2+diabetes
**3**	206157_at	PTX3	PTX3_HUMAN	423	Pentraxin 3 (PTX3)	https://pubmed.ncbi.nlm.nih.gov/?term=PTX3+in+type+2+diabetes
**4**	205943_at	TDO2	T23O_HUMAN	74	Tryptophan 2,3-dioxygenase (TDO2)	https://www.ncbi.nlm.nih.gov/pmc/?term=TDO2+in+type+2+diabetes
**5**	217455_s_at	SSTR2	SSR2_HUMAN	190	Somatostatin receptor 2 (SSTR2)	https://www.ncbi.nlm.nih.gov/pmc/?term=SSTR2+in+type+2+diabetes
**6**	205998_x_at	cyp3a4	CP3A4_HUMAN	2,171	Cytochrome P450 family 3 subfamily A member 4 (CYP3A4)	https://pubmed.ncbi.nlm.nih.gov/?term=Cyp3A4++in+type+2+diabetes
**7**	218313_s_at	GALNT7	GALT7_HUMAN	27	Polypeptide N-acetylgalactosaminyltransferase 7 (GALNT7)	https://www.ncbi.nlm.nih.gov/pmc/?term=GALNT7+in+type+2+diabetes
**8**	202376_at	SERPINA3	G3V3A0_HUMAN	135	Serpin family A member 3 (SERPINA3)	https://www.ncbi.nlm.nih.gov/pmc/?term=SERPINA+3+in+type+2+diabetes

### Cluster analysis

**Figure 3:**
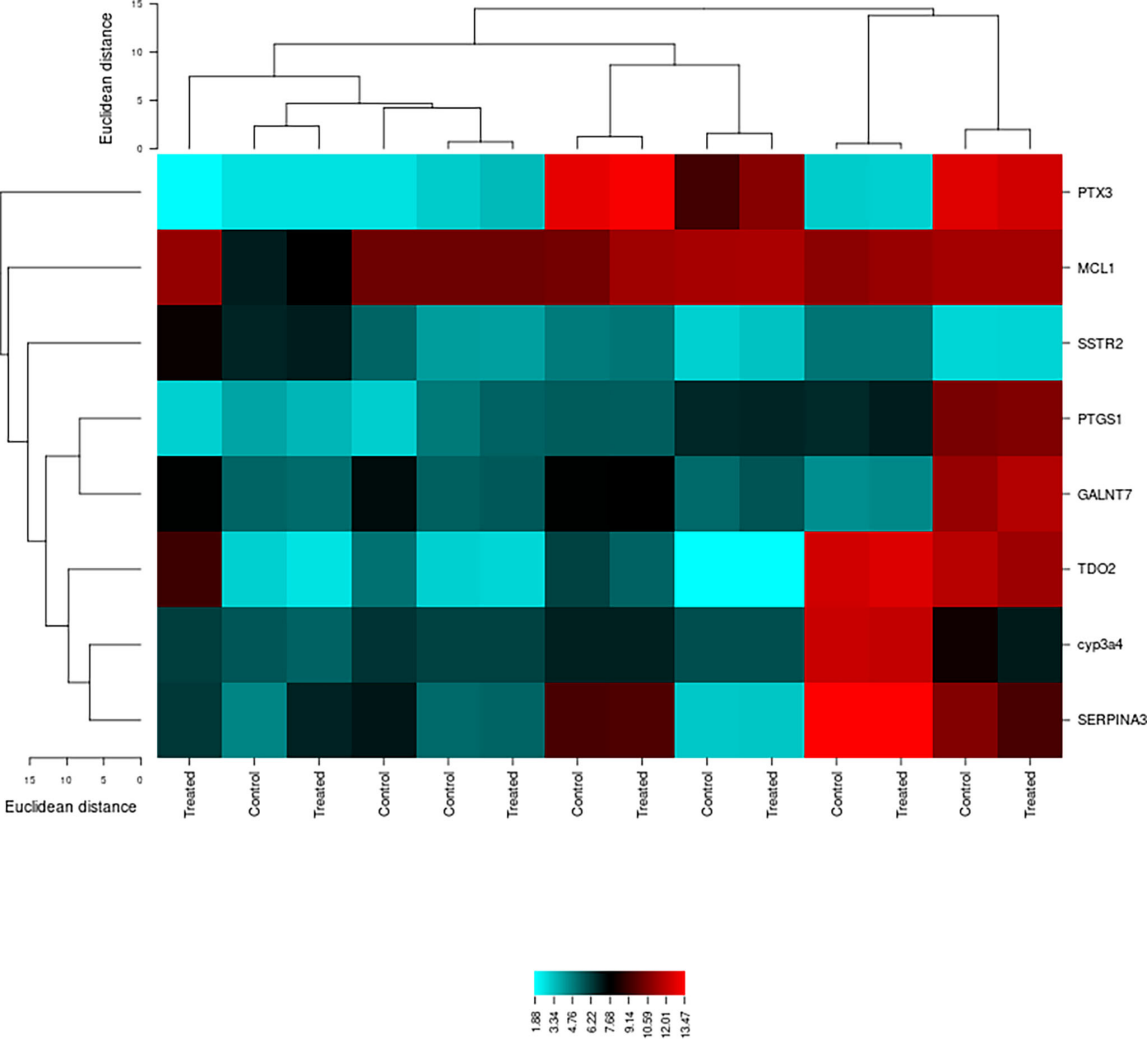
Cluster analysis of type 2 diabetes-associated differentially expressed signature genes using the CIMminer tool. Blue indicates a large distance, while red represents a small distance. Lines show the cluster boundaries in the level of the tree.

The genetic expression of type 2 diabetes samples shows obvious differences among the control and treated samples. **Figure 3** shows the cluster analysis of type 2 diabetes mellitus-related differentially expressed signature genes. The blue color displays a large distance, while the red color indicates a small distance. Lines represent the cluster boundaries at the level of the tree.

### Functional enrichment analysis

**Figure 4:**
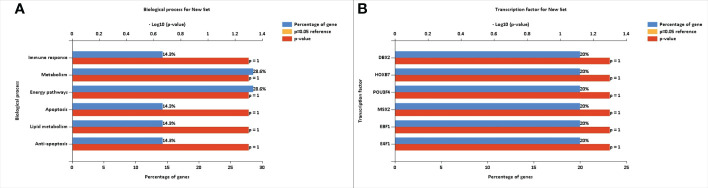
**(A)** Biological processes involved in type 2 diabetes mellitus. **(B)** Transcription factors for type 2 diabetes mellitus-related signature genes that can modify the gene expression in a host cell.

The FunRich tool provided information regarding the percentage of our selected genes that were involved in various biological processes. **Figure 4A** shows the biological processes involved in type 2 diabetes mellitus. The FunRich tool also provided the transcriptional factors that are linked to these differentially expressed genes. The transcription factors identified were DBX2, HOXB7, POU3F4, MSX2, EBF1, and E4F1. The transcription factors (TFs) showed 20% abundance with the known type 2 diabetes mellitus genes. These transcriptional factors also showed a role as targets in type 2 diabetes mellitus. **Figure 4B** shows the transcription factors for type 2 diabetes mellitus-related genes that can modify the genetic expression in a host cell.

DAVID is an online tool that was used to determine the gene ontology and functional annotation of the identified differentially expressed genes. In functional enrichment analysis, the signature genes were short-listed based on fold change and *p*-value cutoff. **Table 3** shows the gene ontology of type 2 diabetes-related DEGs.

**Table 3:** Gene ontology of type 2 diabetes mellitus-associated DEGs.

Category	Term	Count	*p*-value
**GOTERM_BP_DIRECT**	Inflammatory response	3	9.9 × 10^−3^
**GOTERM_BP_DIRECT**	Oxidation–reduction process	3	2.3 × 10^−2^
**GOTERM_BP_DIRECT**	Xenobiotic metabolic process	2	3.2 × 10^−2^
**GOTERM_BP_DIRECT**	Lipid metabolic process	2	6.4 × 10^−2^
**GOTERM_CC_DIRECT**	Organelle membrane	2	3.3 × 10^−2^
**GOTERM_MF_DIRECT**	Heme binding	3	1.3 × 10^−3^
**GOTERM_MF_DIRECT**	Oxygen binding	2	1.9 × 10^−2^

### Network integrome analysis

**Figure 5:**
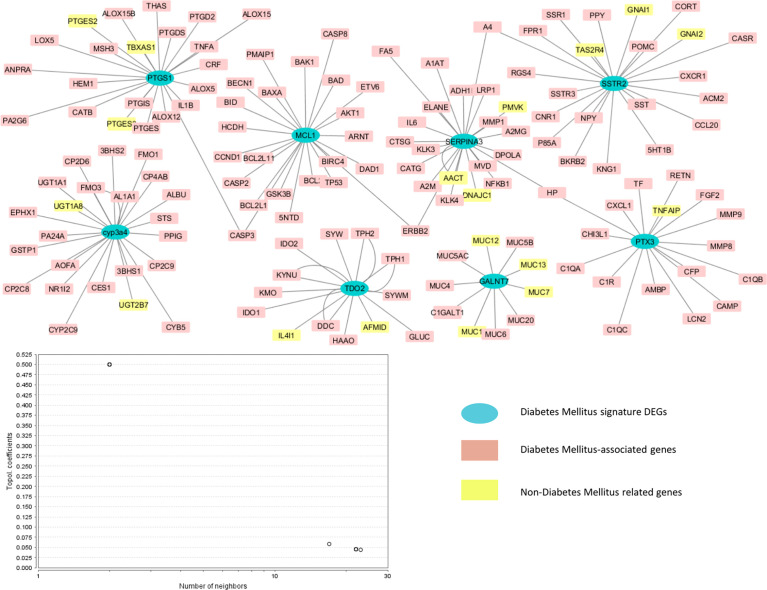
Genetic network of type 2 diabetes-associated differentially expressed signature genes. The blue nodes indicate the type 2 diabetes mellitus-associated potential biomarkers, the yellow nodes represent the non-type 2 diabetes mellitus target proteins, and the pink nodes represent type 2 diabetes mellitus-related DEGs.

The network analysis exposed the interaction of type 2 diabetes mellitus-related signature genes with other genes. The network showed 156 nodes and 158 edges. The network was characterized in three neighborhoods: the blue nodes show the type 2 diabetes mellitus-associated potential biomarkers, the pink nodes show the proteins involved in diabetes mellitus, and the remaining yellow nodes display the non-type 2 diabetes mellitus target proteins. **Figure 5** shows the gene network of type 2 diabetes-related differentially expressed genes.

### miRNA target prediction

Multiple diabetes mellitus-associated miRNA targets for each gene were identified by the computational algorithm miRDB. The miRNAs identified were hsa-miR-3686, hsa-miR-1299, hsa-miR-3163, hsa-let-7a-2-3p, hsa-miR-4306, hsa-miR-4277, hsa-miR-5680, and hsa-miR-296-5p (**Table 4**). The progression and development of diabetes mellitus is linked with the dysregulation of these miRNA targets. The genes *MCL1*, *PTGS1*, *CYP3A4*, *SERPINA3*, *TDO2*, and *GALNT7* predicted 99, 107, 98, 89, 95, and 98 miRNA hits, respectively.

**Table 4:** miRNA targets for diabetes mellitus-associated genes.

UniProt ID	Gene symbol	miRNA	Target score	Total miRNA hits	Structure of predicted miRNA
MCL1_HUMAN	MCL1	hsa-miR-3686	99	188	AUCUGUAAGAGAAAGUAAAUGA
PGH1_HUMAN	PTGS1	hsa-miR-1299	92	107	UUCUGGAAUUCUGUGUGAGGGA
PTX3_HUMAN	PTX3	hsa-miR-3163	91	69	UAUAAAAUGAGGGCAGUAAGAC
T23O_HUMAN	TDO2	hsa-let-7a-2-3p	95	37	CUGUACAGCCUCCUAGCUUUCC
SSR2_HUMAN	SSTR2	hsa-miR-4306	89	62	UGGAGAGAAAGGCAGUA
CP3A4_HUMAN	cyp3a4	hsa-miR-4277	98	69	GCAGUUCUGAGCACAGUACAC
GALT7_HUMAN	GALNT7	hsa-miR-5680	98	197	GAGAAAUGCUGGACUAAUCUGC
G3V3A0_HUMAN	SERPINA3	hsa-miR-296-5p	63	2	AGGGCCCCCCCUCAAUCCUGU

## Discussion

Owing to the heterogeneous nature of type 2 diabetes mellitus and its complex effects on hemodynamics, there is a need to identify new targets to overcome the effects which are involved in the progression of type 2 diabetes mellitus (23). To reduce the worldwide burden of type 2 diabetes mellitus, targeted policies are required to be put into practice with better beneficial efforts. The main objective of this study was to find potential biomarkers for type 2 diabetes mellitus which can later be utilized in antidiabetic therapy.

In this study, the affy package was implemented in the R statistical program and scripting language for several reasons (24). The software is free of cost and is very effective in the differential expression analysis of several genes that can be used as targets for the better management of disease (11, 12). Several gene signatures were identified through the system biology approach, and their functional annotation and protein–protein interactions for a better understanding of type 2 diabetes mellitus progression were performed. The differential expression analysis resulted in 50 differentially expressed genes by pairwise comparison among the physiologically similar groups. From the 50 differentially expressed genes, the topmost 2 genes were graded and nominated from each dataset. The DAVID tool was used to retrieve the gene symbol, UniProt ID, and protein name. Data curation was done to shortlist the genes on the basis of high count by using various databases, i.e., PubMed, PMC, OMIM, and MeSH. The differential study exposed eight potential gene signatures out of the 20 DEGs on the basis of physicochemical and functional evidence, which play a role in type 2 diabetes. *MCL1*, *PTX3*, *CYP3A4*, *PTGS1*, *SSTR2*, *SERPINA3*, *TDO2*, and *GALNT7* were the identified DEGs, out of which seven were upregulated (*MCL1*, *PTX3*, *PTGS1*, *SSTR2*, *SERPINA3*, *TDO2*, and *GALNT7*) and one was downregulated (*CYP3A4*). The cluster analysis was done by using the CIMminer tool that showed variations between the control and treated groups using yellow and blue colors.

The differentially expressed gene *MCL1* belongs to the BCL2 family of antiapoptotic proteins, that is, myeloid cell leukemia sequence 1 (Mcl-1). It is an apoptosis regulator and type 2 diabetes mellitus is triggered by impaired β-cell function. The pro-inflammatory cytokines downregulated the *MCL1* gene causing β-cell apoptosis (25). The role of *MCL1* in diabetes mellitus needs to be characterized. However, due to its restricted role in the control of apoptosis, it can serve as a promising target in the management of diabetes by preventing β-cell apoptosis. The downregulation of *MCL1* is a crucial event in β-cell apoptosis and its role has been studied by Cardozo et al. (26). *PTX3*, another differentially expressed gene, is a pentraxin 3 gene that is involved in the progression of diabetic complications including diabetic nephropathy and retinopathy by the activation of immunological and inflammatory mechanisms (27). The complications of diabetes mellitus are the major obstacles in its treatment including both microvascular and macrovascular complications. Recently, in one study, *PTX3* has been shown to be an accurate marker in revealing diabetic neuropathic progression (28, 29). The involvement of *PTX3* in diabetic complications serves to be an attractive target. Another identified differentially expressed gene, *CYP3A4*, belongs to the cytochrome P450 family, and the presence of these enzymes contributes to low chronic inflammation in type 2 diabetes mellitus (13). The changes in the expression levels of some P450 isoenzymes have shown an association with increased cytokine levels in type 2 diabetes mellitus (14). Induction of *CYP3A4* expression levels has also been observed in hepatic cells due to high serum fatty acid levels clearly showing upregulation of *CYP3A4* in diabetic conditions (15). *PTGS1* is prostaglandin endoperoxide synthase 1 that activates the prostaglandin pathway, and through TNF alpha signaling, the immune system will be upregulated or downregulated and will cause inflammation in type 2 diabetes mellitus (16). The upregulation of the expression of the inflammatory *PTGS* gene in pancreatic islets might be contributing to the dysfunction of islets in diabetes (17). *SSTR2* is somatostatin receptor 2 and inhibits insulin and glucagon release from pancreatic islets by interacting with membrane somatostatin receptors, and it is expressed at high levels on alpha cells and suggested a selective role in the regulation of glucagon release (18). *SERPINA3* belongs to the serpin family A member 3. It increases the trans-endothelial permeability of retinal microvascular endothelial cells and is involved in the pathogenesis of diabetic retinopathy (19). *TDO2* is tryptophan 2,3-dioxygenase and makes hepatic changes in metabolism (20). *GALNT7* is a polypeptide N-acetylgalactosaminyltransferase. It downregulates the expression of type 2 diabetes mellitus (21). The role of the identified differentially expressed genes in the development of diabetes mellitus and their association with the development of diabetic complications have opened new insights for the better management of this disease.

The FunRich software was used to determine the transcriptional factors and biological processes involved in diabetes mellitus. The transcriptional factors identified were DBX2, HOXB7, POU3F4, MSX2, EBF1, and E4F1. The TFs showed 20% abundance with the known type 2 diabetic genes. The identified transcriptional factors also showed a significant role in type 2 diabetes mellitus, serving as targets in the development of new treatment strategies. The biological processes identified were immune response, metabolism, energy pathways, apoptosis, lipid metabolism, and anti-apoptosis. The DAVID tool was used to determine gene ontology. The gene ontology of these genes showed inflammatory response, oxidation–reduction process, xenobiotic metabolic process, lipid metabolic process, organelle membrane, heme binding, and oxygen binding. The role of the identified differentially expressed genes was extensively studied opening new paths in the better management of diabetes mellitus.

The gene network analysis was performed in order to analyze the interaction of the identified seeder genes with other proteins that may be involved in the progression of diabetes mellitus. The HAPPI and STRING databases were used to determine the proteins associated with each other and other proteins. The Cytoscape software was used to visualize the network. The role of target proteins in type 2 diabetes was further confirmed by using various databases, i.e., PubMed, PMC, OMIM, and MeSH. The databases identified the proteins having a role in diabetes mellitus (colored in red and pink) and also the non-diabetes-associated proteins (colored in yellow). Several interacting proteins that showed association with *MCL1* were BCL2, BAD, CASP2, CCND1, TP53, etc. *PTX3* showed association with TNFAIP, MMP9, CXCL, CAMP, and TF; *CYP3A4* with FMO1, UGT, and CYB5; and *PTGS1* with ALOX1, PTGE, LOX5, HEM1, MSH3, and TNFA. *SSTR2* showed interactions with NPY, POMC, SST, and SSR1. The identified signature genes and the interacting proteins have shown roles in the development of diabetes mellitus. The interaction analysis has shown how these potential biomarkers interact with each other as well as with other proteins in causing diabetes mellitus. The literature has also confirmed their role in the progression of type 2 diabetes mellitus; hence, these genes can further be used as potential targets to treat diabetes mellitus by formulating drugs that would specifically target these genes. The secondary genes *TNFAIP*, *MMP9*, and *CXCL* showing interaction with *PTX3* have a potential role in diabetes mellitus (22, 30, 31). Several lines of evidence have also supported the role of the secondary genes *FMO1* and *ALOX1* having interaction with *CYP3A4* and *PTGS1* in diabetes mellitus (31, 32). Most of the signature genes that do not show a role in diabetes mellitus provided evidence of the contribution of these genes interacting with DEGs in causing diabetes mellitus. In conclusion, the study has identified the potential candidate biomarkers for diabetes mellitus that are recently emerging as new targets in disease management, and the interaction of these genes with other proteins through network analysis has further clarified the role of these genes in the progression of diabetes mellitus.

## Conclusion

The study has helped us in identifying candidate biomarker genes for diabetes mellitus using differential expression analysis. *MCL1*, *PTX3*, *CYP3A4*, *PTGS1*, *SSTR2*, *SERPINA3*, *TDO2*, and *GALNT7* were the identified DEGs. Several lines of evidence have shown the role of these genes in the development of diabetes mellitus. Moreover, the study has also explored the interaction of these genes with other proteins involved in crosstalks with these genes. The crosstalks between these genes are responsible for the progression and the complications associated with diabetes mellitus. The gene ontology and functional annotation analysis have also helped us in understanding the biological processes, pathways, and transcriptional factors associated with these genes. The study has opened new insights related to the role of these DEGs in diabetes mellitus, and targeting these genes could potentially help us in the better management of this disease.

## Data availability statement

The datasets presented in this study can be found in online repositories. The names of the repository/repositories and accession number(s) can be found in the article/**Supplementary Material**.

## Author contributions

Conceptualization: TH, UI, RA, and MA. Writing—original draft preparation: BN, UI, RA, and MA. Writing—review and editing: BZ, HZ, AP-S, NN, and MI. Supervision: UI, MA, and YD. All authors have read and agreed to the published version of the manuscript.

## Funding

This study was financed by Riphah International University (Riphah-ORIC-21-22/FPS-51 and Riphah-ORIC-21-22/FPS-56).

## Conflict of interest

The authors declare that the research was conducted in the absence of any commercial or financial relationships that could be construed as a potential conflict of interest

## Publisher’s note

All claims expressed in this article are solely those of the authors and do not necessarily represent those of their affiliated organizations, or those of the publisher, the editors and the reviewers. Any product that may be evaluated in this article, or claim that may be made by its manufacturer, is not guaranteed or endorsed by the publisher.

## Supplementary material

Additional file 1 Table S1: A single summary statistic for each array provided the function summary AffyRNAdeg of Bioconductor package in each dataset.

Additional file 2 Table S2: Databases, software, and tools used in the study.

Additional file 3 Table S3: Initial examination of common and related differential genes of each microarray dataset.
